# Live imaging looks deeper

**DOI:** 10.7554/eLife.30515

**Published:** 2017-09-04

**Authors:** Tanner C Fadero, Paul S Maddox

**Affiliations:** 1 Biology DepartmentUniversity of North Carolina at Chapel HillChapel HillUnited States

**Keywords:** Planaria, confocal microscopy, refractive index matching, live-imaging, organoids, Human, Planarian, Zebrafish

## Abstract

Iodixanol provides an easy and affordable solution to a problem that has limited resolution and brightness when imaging living samples.

**Related research article** Boothe T, Hilbert L, Heide M, Berninger L, Huttner WB, Zaburdaev V, Vastenhouw NL, Myers EW, Drechsel DN, Rink JC. 2017. A tunable refractive index matching medium for live imaging cells, tissues and model organisms. *eLife* **6**:e27240. doi: 10.7554/eLife.27240

Microscopes rely on lenses bending, or refracting, light in predictable ways to allow us to look at objects that are otherwise too small to see. Yet, for as long as scientists have used lenses to magnify and observe life, problems have occurred when light does not refract as predicted. Any departure from the norm is called an aberration, and modern imaging systems have complex arrays of lenses with specialized coatings to limit most optical aberrations. However, these highly engineered systems are designed to optimally focus on objects at a fixed distance, typically the surface of the coverslip placed over the sample on a microscopy slide (Cargille, 1985). Focusing any deeper into samples within a watery solution – like a living cell or tissue – raises problems once again because of a phenomenon known as spherical aberration.

Light travels faster through water than it travels through glass or most biological samples. The speed of light in a given material is described by a property called its refractive index; and the higher the refractive index the slower light will travel. Spherical aberration occurs when light from an object – such as a fluorescently tagged protein – crosses the boundary between two materials with different refractive indices – for example, the biological sample and the surrounding solution – at an angle, and then bends because it changes speed. As the sample moves deeper into an aqueous solution, the point where the light intersects with the coverslip moves as well, and further bending by refraction can prevent the light from being captured by the lens (Figure 1). This effectively decreases both the resolution and brightness of the image, making it too blurry and too dim to distinguish meaningful features of objects further into the sample.

**Figure 1.**
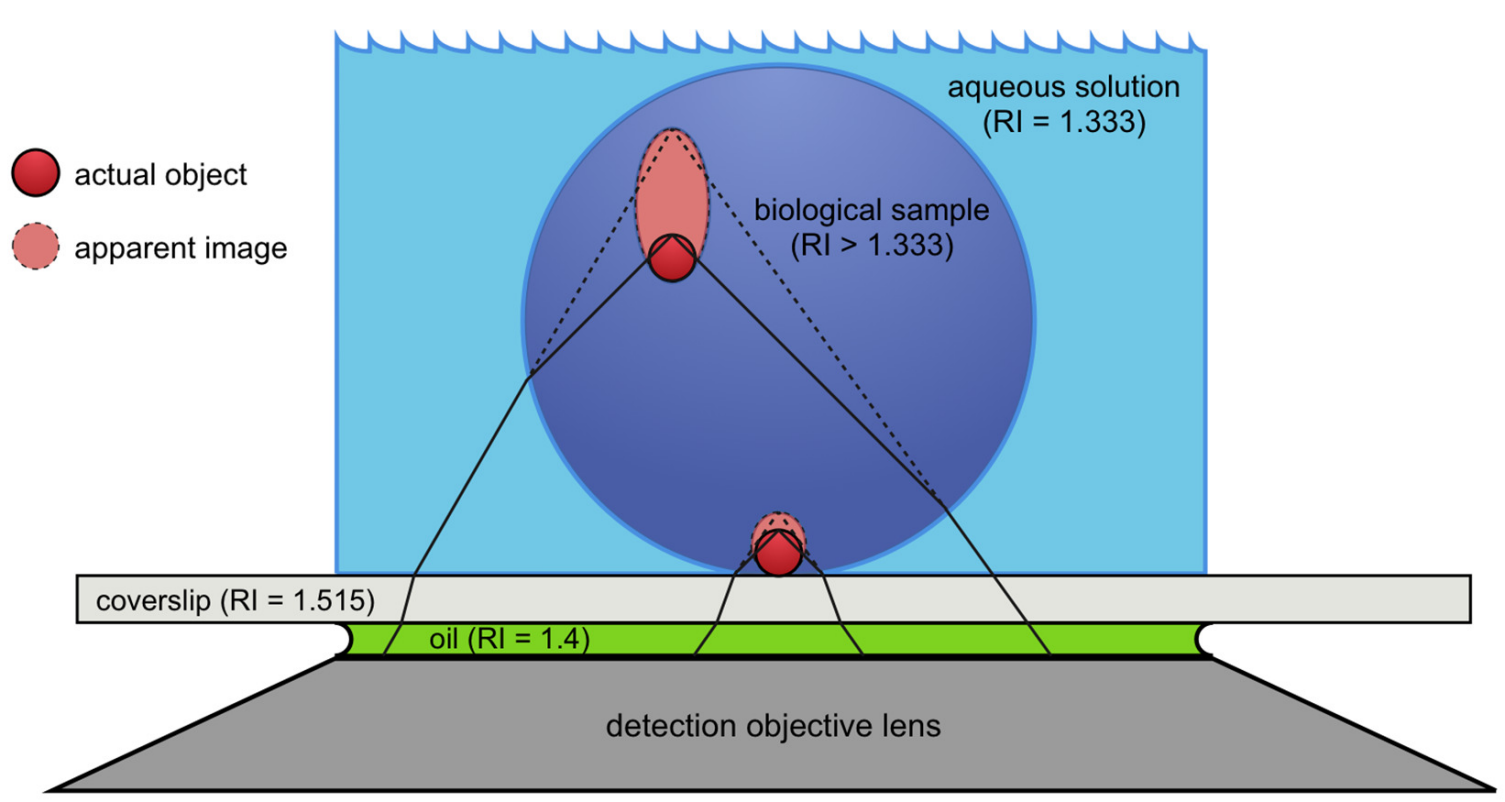
Spherical aberration causes image distortion in fluorescence microscopy. Actual fluorescent objects (red circles) emit light (solid black lines) that is collected by the detection objective lens (dark gray trapezoid). The emitted light bends (refracts) at the boundary between the biological sample (dark blue circle) and the aqueous solution (light blue) because the refractive index (RI) is different in each material. The refraction causes the light to appear to be coming from a different location (dashed lines) than the actual object, creating an apparent image (light red oval) that is distorted. The further away from the coverslip (light gray rectangle) the object is, the more distorted its apparent image becomes. Oil (green) is used between the coverslip and the lens to obtain images with higher resolution.

Biologists looking at living samples and wanting to see deeper than about 10 micrometers from the surface have previously needed microscopes with lower resolution, corrective adjustments, or 'adaptive optics systems' to minimize the effects of spherical aberration (Booth, 2007). These technologies, however, have limited practicality and are often expensive. Now, in eLife, Jochen Rink of the Max Planck Institute of Molecular Cell Biology and Genetics and colleagues report a simpler and more affordable approach (Boothe et al., 2017).

For non-living – or fixed – specimens, the problem of spherical aberrations has long been overcome by replacing the water with an optically clear substance with a high refractive index to better match that of glass. Yet many of the substances currently used, such as glycerol, are toxic to living samples. Rink and colleagues – including Tobias Boothe as first author – instead looked for a water-soluble compound with a high refractive index that was not toxic. A compound called iodixanol met all their requirements and they showed that when added to the surrounding solution at the proper concentration the biological sample effectively became 'invisible'. This occurred because light from the object did not experience a change in refractive index when it traveled between the sample and the solution, which meant that fluorescent objects within could be seen more clearly. No change in refractive index meant that the light was no longer refracted when it exited the sample. In other words, spherical aberration was greatly reduced.

Boothe et al. demonstrate the benefits of decreasing the spherical aberration in live samples by imaging deep into developing zebrafish embryos and planarian flatworms. Fluorescent markers in animals mounted in a solution containing iodixanol looked sharper and brighter than those in a more traditional aqueous solution. As would be expected, the improvements in optical resolution and brightness were more pronounced for objects at greater depths away from the coverslip.

Boothe et al. confirm that iodixanol is compatible with living samples by showing that various zebrafish embryos, human cell cultures and planarian flatworms can develop, proliferate, and even regenerate in the presence of high concentrations of the substance. This method represents a breakthrough for scientists looking to obtain high-quality images from living organisms. Microscopists will, however, still face challenges in matching the refractive index of the surrounding solution to the sample, because most organisms consist of multiple materials of different refractive indices. As such, the technique presented by Boothe et al. is a step forward for the field, but opportunities remain to further improve image quality in complex organisms.
